# Habitat diversification associated with urban development has a little effect on genetic structure in the annual native plant *Commelina communis* in an East Asian megacity

**DOI:** 10.1002/ece3.10975

**Published:** 2024-02-21

**Authors:** Nakata Taichi, Naoyuki Nakahama, Nobuko Ohmido, Atushi Ushimaru

**Affiliations:** ^1^ Graduate School of Human Development and Environment Kobe University Kobe Japan; ^2^ Institute of Natural and Environmental Sciences University of Hyogo Sanda Japan; ^3^ Museum of Nature and Human Activities Hyogo Japan

**Keywords:** Asian dayflower, *Commelina communis*, genetic diversity, isolation by distance, MIG‐seq, urbanisation

## Abstract

Urban development greatly alters the natural and semi‐natural habitats of native plants. Urbanisation results in a range of diverse habitats including remnant agricultural lands, urban parks, and roadside habitats. This habitat diversity often promotes trait divergence within urban areas. However, the mechanisms by which diverse urban habitats influence the population genetic structure of individual plant species remain poorly understood. We investigated the effects of urbanisation on genetic diversity and structure within 24 *Commelina communis* populations across diverse habitat types (rural agricultural land, urban agricultural land, urban park land, and urban roadsides) within the Kyoto–Osaka–Kobe megacity in Japan. We conducted multiplexed inter‐simple sequence repeat genotyping to compare genetic diversity among populations in different habitats. We also examined the correlation between Nei's genetic distance and geographic and environmental distances and performed principal coordinate analysis (PCoA) to evaluate genetic differentiation among urban habitats. There were no significant differences in genetic diversity indices between urban and rural populations and among urban habitat types. Although we detected no isolation‐by‐distance structure in population pairs of the same habitat type and in those of different habitats, the difference in surrounding landscape facilitated genetic differentiation not only between urban and rural habitats but also between different urban habitats. PCoA revealed no clear genetic differentiation among rural and urban habitat populations. Our findings indicate that the establishment of diverse habitat types through urbanisation has no and little impact on genetic diversity and structure, respectively, in *C. communis*, likely due to its high selfing rate and ability to adapt to urban conditions.

## Graphical abstract

Our findings indicate that the establishment of diverse habitat types through urbanisation has no significant impact on genetic diversity and only a marginal effect on genetic structure in *Commelina communis*. This is likely attributed to its high selfing rate and its capacity to adapt to urban conditions.

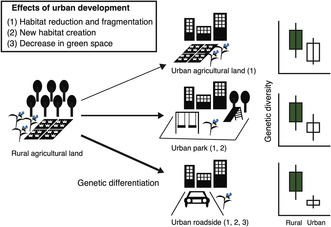

## 1 INTRODUCTION

Anthropogenic habitat changes caused by urban development have significantly impacted the habitats of plants and animals in natural and semi‐natural ecosystems (Johnson & Munshi‐South, 2017; Sukopp, 2004; Williams et al., 2009, 2015). Such changes are generally associated with biodiversity loss (Aronson et al., 2017; McKinney, 2008; Uchida et al., 2018) but also provide opportunities for rapid adaptation by organisms to novel environments (Johnson & Munshi‐South, 2017; Rivkin et al., 2019; Thompson et al., 2022). Habitat reduction and fragmentation often cause decreases in population size and isolation from other populations, which can lead to bottleneck effects, limited interpopulation gene flow, and/or genetic drift (Cheptou et al., 2017; Miles et al., 2019, 2021). In some plant species, habitat reduction and fragmentation have acted as selective pressures driving trait evolution towards more limited dispersal in cities (Cheptou et al., 2008; Dubois & Cheptou, 2017).

It is crucial to understand the population genetic structure of a target organism to be able to examine its evolutionary trends within cities, because artificial fragmentation effects vary among species (Cheptou et al., 2017). Urbanisation‐induced habitat reduction and fragmentation are generally thought to lead to lower genetic diversity within populations and higher genetic differentiation among populations (Miles et al., 2019). Focused on vascular plants, some studies have suggested reduced gene flow among urban populations (Bartlewicz et al., 2015; Dornier & Cheptou, 2012; Emel et al., 2021; Nagamitsu et al., 2014; Noreen et al., 2016), whereas others have reported that urban fragmentation does not lead to reduced gene flow or connectivity among populations (Breinholt et al., 2009; Culley et al., 2007; Roser et al., 2017; Van Rossum, 2008; Wang et al., 2019). Thus, the results differ depending on species, making it difficult to draw general conclusions.

**FIGURE 1:**
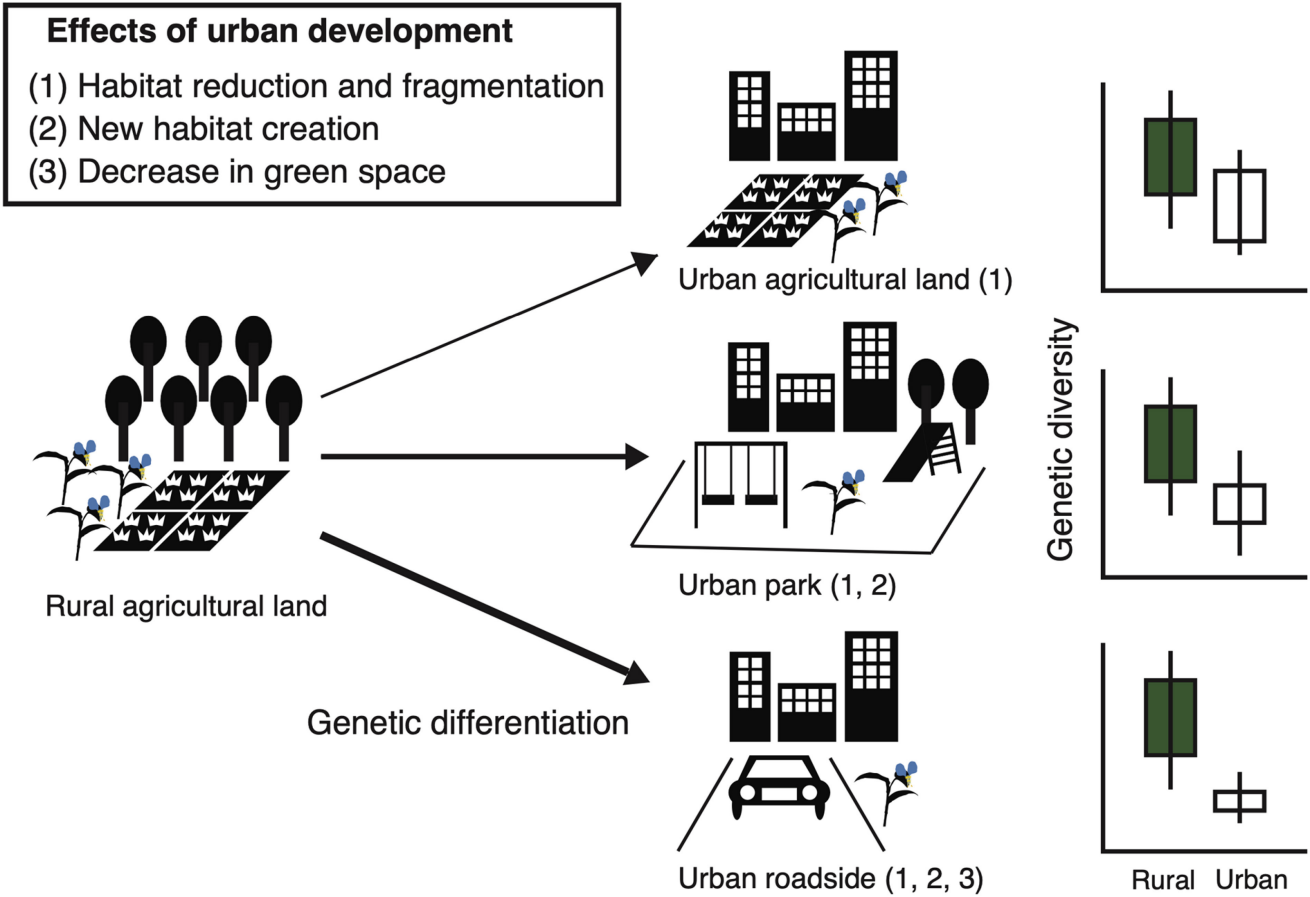
The conceptual figure illustrates the predictions of this study. We hypothesised that populations in urban agricultural lands would experience reduced genetic diversity due to the impact of habitat reduction and fragmentation associated with increased developed land area (1). In addition to the habitat changes, the genetic diversity would be lower in urban park populations which establish on newly created habitats (1, 2). The reduction in surrounding green areas would further contribute to the loss of genetic diversity in urban roadside populations (1, 2, 3). The width of the arrows indicates the degree of genetic differentiation.

Although previous urban studies have compared genetic structures between urban and rural populations (Johnson et al., 2018; Rivkin & Johnson, 2022; Yakub & Tiffin, 2017), genetic differences among diverse urban habitats have rarely been examined. Urban areas usually consist of various habitat types, such as remnant agricultural land, park land, and roadsides, which significantly differ in their environmental conditions and habitat configurations (Baldock et al., 2019; Vakhlamova et al., 2022). Recent studies have emphasised the importance of within‐city habitat diversity, which promotes phenotypic trait differentiation across habitat types (Kostanecki et al., 2021; Santangelo, Roux, & Johnson, 2022; Woudstra et al., 2023). Among species that initially inhabit agricultural environments, populations in urban agricultural lands are likely influenced by habitat reduction and fragmentation through increasing development, whereas populations in urban parks and roadsides are likely established through immigration to smaller newly created habitats that are already highly fragmented. Barochorous annual and perennial species with short life cycles often invade into these newly created habitats, such as urban parks (Iwachido et al., 2023) and roadside cracks (Hayasaka et al., 2012). Lower genetic diversity may be found in urban park and roadside populations than in remnant agricultural lands, due to founder effects and smaller habitat size. Furthermore, compared to roadsides, urban parks are expected to have greener habitat areas. Consequently, the extent of population size reduction and/or isolation is more pronounced for roadside habitats, followed by urban parks and residual agricultural areas. Thus, differences in genetic diversity and genetic differentiation among urban populations may be predicted for different habitat types (Figure 1).

We tested these predictions by examining populations of the annual native plant *Commelina communis* in rural agricultural areas and three urban habitats (urban agricultural land, parks, and roadsides) in a megacity of Japan. This species is widely distributed in agricultural and ruderal habitats along an urban–rural gradient characterised by phenotypic variation in floral traits (Ushimaru et al., 2014). We investigated whether all three urban population types exhibit lower genetic diversity and more limited gene flow than rural populations, and whether genetic diversity and the degree of genetic differentiation differ among urban habitat types. Our findings provide insights that will be useful for designing population maintenance strategies to maintain the genetic diversity of this plant species in urban environments.

## 2 MATERIALS AND METHODS

### 2.1 Study systems

*Commelina communis* (Commelinaceae) is an annual native plant that is used as a model species to compare genetic diversity and structure between rural and urban habitat populations. This species naturally thrives in temperate regions of northeastern Asia and is commonly found in paddy fields, parks, and roadside areas. Each plant typically produces multiple inflorescences with perfect and staminate flowers that open at sunrise and close around noon each day. This species is self‐compatible, and its perfect flowers exhibit autonomous self‐pollination (Katsuhara & Ushimaru, 2019; Morita & Nigorikawa, 1999) as well as reproductive traits (the presence of staminate flowers, large blue petals, and rewarding yellow anthers) that promote outcrossing (Ushimaru et al., 2007, 2009).

Our study area consisted of 25 sites selected within the Kyoto–Osaka–Kobe metropolitan area (34°672′–35°111′ N, 135°219′–822′ E, Figure S1), one of the world's largest megacities with approximately 19 million residents (United Nations, 2019). Within the megacity, paddy fields and forests on flat land have faced habitat loss and fragmentation due to rapid urbanisation since the 1980s (Saizen et al., 2006; Tsuji et al., 2011). In 2021–2022, we collected leaf samples from each of 185 *C. communis* individuals at 25 sites, including eight rural agricultural (RA), six urban agricultural (UA), five urban park (UP), and six urban roadside (UR) sites, in Kyoto, Osaka, and Hyogo Prefecture, Japan. The geographical distances between the sites ranged from 0.82 to 65.54 km (Figure S1), and those between pairs of UA populations (excluding population *i*) were significantly closer than those between pairs of RA populations (Figure S2).

We calculated the total areas of developed lands, forests, agricultural lands, and grasslands within a 250 m, 500 m, and 1 km radius from the centre of each site to examine the effect of the areas of developed lands and green spaces around the study site on genetic diversity of and genetic differentiation between study populations using high‐resolution land use and land cover map (2018–2020) made by Japan Aerospace Exploration Agency (JAXA, 2020).

In 2021, we collected seeds from 24 sites, including 8 RA, 5 UA, 5 UP, and 6 UR sites (Table S1). At each site, seeds were collected from 1 to 15 individual maternal plants, with an average of six plants per site, each separated by at least 1 m, at each of the 24 collection sites (Figure S1, Table S1). Seeds collected from each maternal plant were stored separately at room temperature. In April 2022, the collected seeds underwent a 2‐month cold and moisture treatment and then were germinated in plastic pots filled with a mixture of red volcanic akadamatuchi soil and vermiculite (Kohnan Shoji Co., Ltd., Japan), which contained no fertiliser. Then the plastic pots were transferred to a greenhouse at Kobe University (34°73′ N, 135°23′ E) and watered daily. A leaf sample was collected from each cultivated individual (from different mother plants) in September 2022. For populations whose seeds did not germinate and those with insufficient leaf samples from cultivated individuals, we conducted additional leaf sampling in the field in September 2022. For each study population, we collected 3–7 leaf samples (mean: 4.6 samples) from different individuals (Table S1). The collected leaf samples were rapidly dried in silica gel at room temperature (15–30°C).

### 2.2 Molecular data collection and sequencing analysis

We extracted genomic DNA from dried leaf samples using the DNeasy Plant Mini Kit (Qiagen, Hilden, Germany). We performed multiplexed inter‐simple sequence repeat (ISSR) genotyping (MIG) analysis to detect genome‐wide single‐nucleotide polymorphisms (SNPs) (Suyama & Matsuki, 2015) under standard experimental conditions (Suyama et al., 2022; Suyama & Matsuki, 2015). We amplified ISSRs from the genomic DNA using eight pairs of multiplex ISSR primers that were developed as MIG primers. We incubated 5 μL polymerase chain reaction (PCR) products with 3.5 μL AMPureXP beads (Beckman Coulter, Tokyo, Japan); the product was diluted 50 times with deionised water to remove short DNA fragments (<200 bp) and used as template DNA for a second PCR. To construct an indexed library suitable for Illumina sequencing, each sample was uniquely prepared. The combined PCR products (1 μL each) constituted a single library, which was subjected to purification and size selection (400–800 bp) using the Pippin Prep DNA size selection system (Sage Science, Beverly, MA, USA). The DNA concentration of the size‐selected libraries was measured using a SYBR green quantitative PCR assay (Library Quantification Kit, Takara Bio, Shiga, Japan), using primers specifically designed for the Illumina constructs. Sequencing was conducted using an Illumina MiSeq system (Illumina, San Diego, CA, USA) with the MiSeq Reagent Kit v3 (150 cycles, Illumina) at a final library concentration of 12 pM. During data pre‐processing, we removed low‐quality reads and primer sequences from the raw data using the Trimmomatic version 0.39 (Bolger et al., 2014), following the approach of Suyama et al. (2022).

We de‐multiplexed the quality‐filtered sequence data and filtered the resulting data using Stacks v2.59 (Catchen et al., 2011, 2013). The *ustacks* program was run with the following parameter settings: minimum coverage depth to create a stack (*m*) = 3, the maximum distance between stacks (*M*) = 2, maximum distance to align secondary reads to primary stacks (*N*) = 2; the deleveraging (*d*) and removal (*r*) algorithms were enabled. Subsequently, we employed the *cstacks* program, setting the number of mismatches allowed between sample loci during catalogue building (*n*) to 2. The catalogue was searched using the *sstacks* program. Then, we applied the *populations* program with the following parameter settings: minimum proportion of individuals required to process a locus across all data (*r*) = 0.8, data analysis restricted to a single SNP per locus (write_single_snp), the minimum number of populations per locus (*p*) = 1, minimum minor allele frequency required to process a nucleotide site at a locus (min_maf) = 0.01, and maximum observed heterozygosity required to process a nucleotide site at a locus (max_obs_het) = 0.95. Genetic data from population *i*, which had a high proportion of missing data (>40%), were excluded from subsequent statistical analyses.

We also compared population genetic statistics using different combinations of Stacks settings (Weiss et al., 2018), because a previous study suggested that *C. communis* is polyploid (Morita & Nigorikawa, 1999). Despite a lack of differences in the population genetic statistics (Table S2), a high number of SNPs was detected; therefore, we decided to adopt the Stacks settings listed above. The resulting data were used in our subsequent analyses.

### 2.3 Comparisons of genetic diversity and structure

We conducted the Hardy–Weinberg test by using GenoDive v3.06 to assess whether random mating occurs within each population (Meirmans, 2020).

By using R v4.22 (R Core Team, 2022), we evaluated the allelic richness (*A*_R_; El Mousadik & Petit, 1996), observed heterozygosity (*H*_O_), expected heterozygosity (*H*_E_), and inbreeding coefficient (*F*_IS_) using *basic.stats* function in the *hierFSTAT* package (Goudet, 2005) to estimate genetic diversity. The target number of rarefied alleles was set to 5 for *A*_R_ evaluation. We also evaluated the average number of alleles per locus (N_A_) using GenAlEx v6.41 (Peakall & Smouse, 2006). We compared these genetic diversity metrics (*N*_A_, *A*_R_, *H*_O_, *H*_E_, and *F*_IS_) among habitat types using a generalised linear model (GLM) with Gaussian error distribution and an identity link. To assess the effects of surrounding landscape configuration on the genetic diversity of study populations, we also used a GLM with a Gaussian distribution and identity link. Each genetic diversity metric, total areas of developed lands, agricultural lands, and forests around each site at either of three different spatial scales were the response variable, and explanatory variables, respectively. We also examined a null model without landscape variables for each metrics. We selected the lowest Akaike information criterion model with landscape variables of a certain spatial scale as the best model.

To assess genetic differentiation at the population level, we calculated the pairwise Nei's genetic distance and the fixation index (*F*_ST_) as indices of genetic divergence among the 24 populations using GenAlEx v6.41 (Peakall & Smouse, 2006). A Mantel test detected a significantly positive correlation between the two indices (Figure S3, Mantel statistic *r*: .967, *p* < .001); therefore, we adopted Nei's genetic distance as the genetic differentiation indicator for this study. We assessed the genetic relationships among populations within each habitat type through principal coordinate analysis (PCoA) using GenAlEx v6.41 (Peakall & Smouse, 2006). To compare within‐habitat genetic differentiation between habitat types, we used the *vegan* package in R (Oksanen et al., 2007) to perform a Mantel test (Mantel, 1967), which allowed us to analyse pairwise Nei's genetic distances using geographic distance as a covariate for up to 1000 permutations. Because the *vegan* package cannot treat isolation‐by‐distance relationships between pairs of different habitat types, we compared correlations between genetic and geographic distances between population pairs of different habitat types by performing 1000 permutations and estimating 95% confidence intervals (CIs).

Next, we calculated the pairwise landscape difference between a pair of populations based on the Bray–Curtis distance of surrounding landscape (differences in developed and agricultural land and forest areas) and its effect on genetic differentiation by using a *vegan* package (Oksanen et al., 2007) in the same way as in the isolated‐by‐distance analyses described above using the Mantel test.

We further compared Nei's genetic distances between urban and rural habitat pairs, between urban habitat pairs, and between all population pairs (ALL). One pair was randomly resampled from each habitat pair type and the resampling procedure was repeated 1000 times to estimate the means and 95% CIs for each set of pairs. These statistical analyses were performed using R.

## 3 RESULTS

### 3.1 Genetic diversity of each habitat type

The Hardy–Weinberg test showed that all populations exhibited significant excesses of homozygotes, and 92.6% of SNPs (562/607 loci) have significantly deviated from the Hardy–Weinberg equilibrium (Table S1).

**FIGURE 2:**
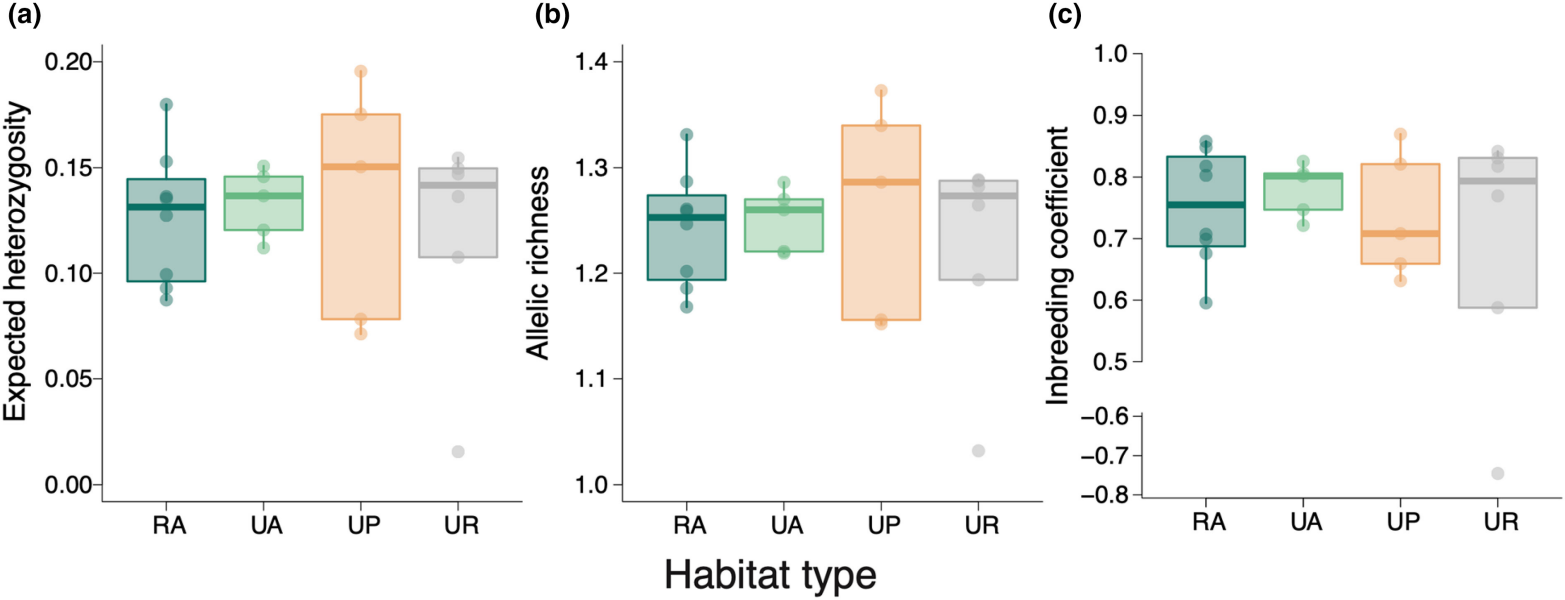
Comparisons of genetic diversity parameters for *Commelina communis*, including the (a) expected heterozygosity, (b) allelic richness, and (c) inbreeding coefficient, among four habitat types. Bold horizontal lines in boxplots indicate medians. Each dot represents a single population. RA, rural agricultural land; UA, urban agricultural land; UP, urban park; UR, urban roadside.

We estimated the parameters *H*_O_, *H*_E_, *N*_A_, *A*_R_, and *F*_IS_ to assess the effects of urbanisation on genetic diversity (Table S1). The dataset included 607 SNPs, with an 83.0% coverage rate for 180 individuals. None of the parameters significantly differed among habitat types (Figure 2, Figure S4, Table S3). The null models without landscape variables were the best models for all genetic metrics and landscape variables had no significant effects on genetic diversity metrics at all spatial scales (Table S3).

### 3.2 Genetic differentiation among urban and rural populations

**FIGURE 3:**
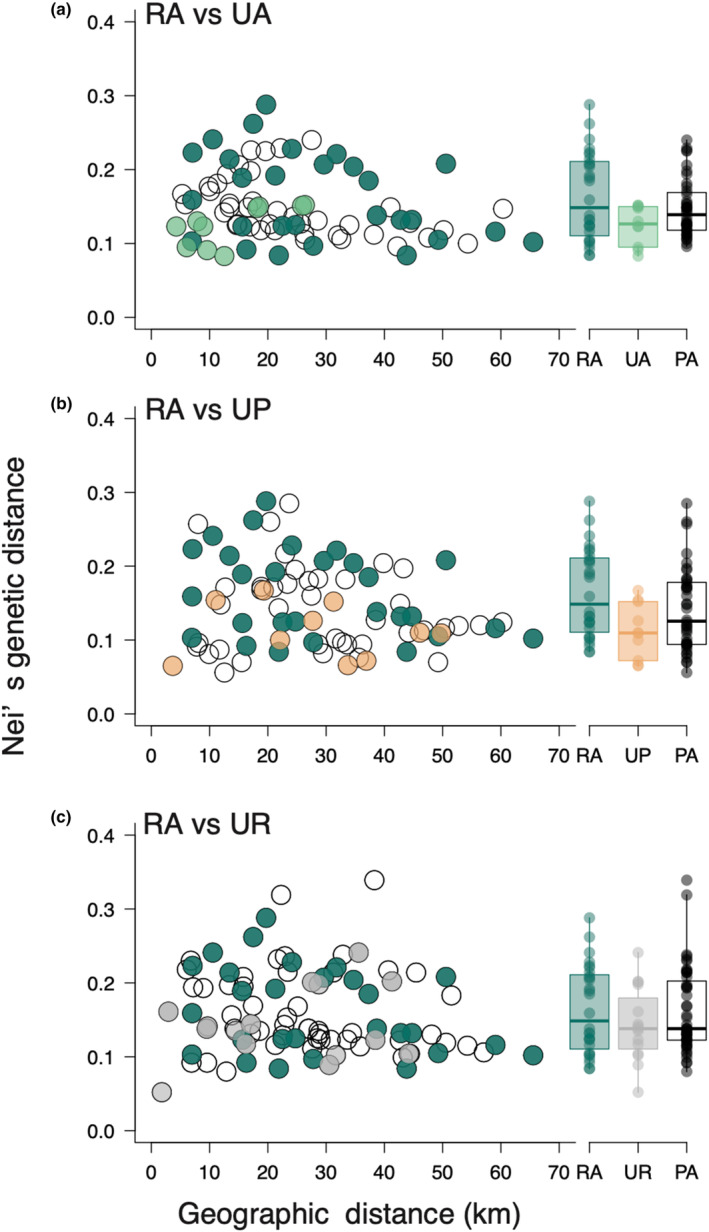
Relationships between Nei's genetic distance and geographic distance (km) for (a) RA–UA, (b) RA–UP, and (c) RA–UR population pairs. PA, all populations. Bold horizontal lines in boxplots indicate medians. Each dot represents a single population. RA, rural agricultural land; UA, urban agricultural land; UP, urban park; UR, urban roadside.

We investigated whether urban development and urban habitat‐type diversity affected the relationships of genetic differentiation with geographic distance and landscape difference. Within‐habitat genetic distance was not dependent on geographic distance in any habitat type (Figure 3; for RA population pairs, Mantel statistic *r* = −.328, *p* = .955; for UA pairs, Mantel statistic *r* = .677, *p* = .192; for UP pairs, Mantel statistic *r* = −.116, *p* = .633; for UR pairs, Mantel statistic *r* = .295, *p* = .132). There were no significant negative correlations between genetic and geographic distance among the RA_UA pairs or among the RA_UP and RA_UR pairs (Figure 3, Figure S5a; for RA_UA pairs, *r* = .438; for RA _UP pairs, *r* = −.141; for RA_UR pairs, *r* = −.146). For urban habitats, we detected significant positive correlations between genetic and geographic distance among UA_UR pairs (Figure S5b; UA_UR pairs, *r* = .550; for UA_UP pairs, *r* = −.235; for UP and UR pairs, *r* = −.046).

**FIGURE 4:**
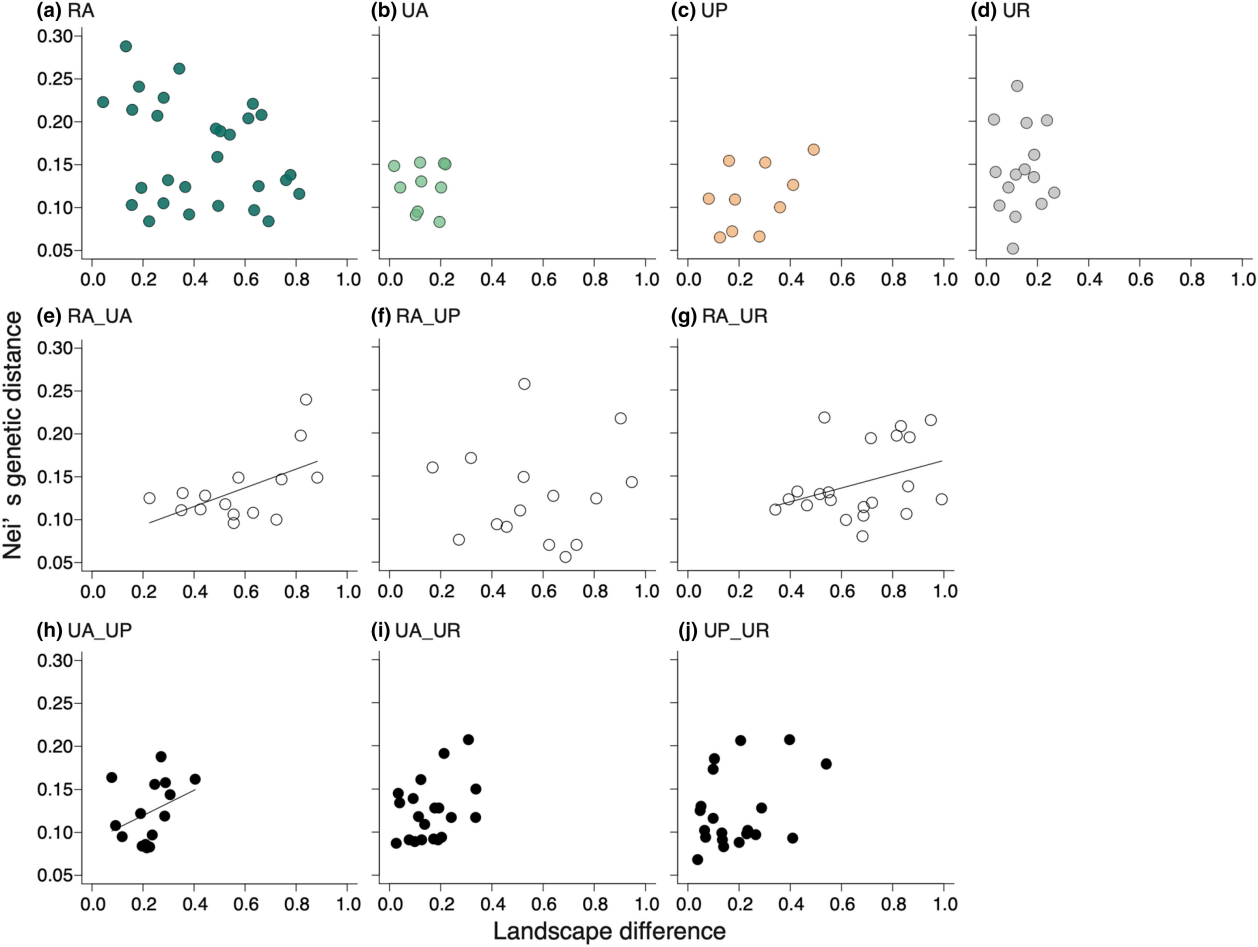
Relationships between Nei's genetic distance and landscape difference for (a) RA, (b) UA, (c) UP (d) UR, (e) RA_UA, (f) RA_UP (g) RA_UR, (h) UA_UP, (i) UA_UR, and (j) UP_UR population pairs. Regression lines were drawn using the estimated coefficients from the general linear model analyses.

Genetic distance between all population pairs had the highest correlation with their landscape differences within the radius of 500 m from the study site (250 m: *r* = .252, *p* = .017; 500 m: *r* = .3066, *p* = .021; 1 km: *r* = .267, *p* = .018). We therefore performed all subsequent analyses using the 500 m scale landscape data. Within‐habitat genetic distance was not influenced by landscape difference in all habitat types (Figure 4a–d; for RA pairs, *r* = −.299, *p* = .956; for UA pairs, Mantel statistic *r* = .018, *p* = .342; for UP pairs, *r* = .457, *p* = .125; for UR pairs, *r* = .044, *p* = .474). There were significant positive correlations between genetic distance and landscape difference for the RA_UA and RA_UR pairs, but not for the RA_UP pairs (Figure 4e–g; for RA_UA pairs, *r* = .455, 95% CIs, −0.328 – 0.312; RA_UP pairs, *r* = .212, 95% CIs, −0.318 – 0.330; RA_UR pairs, *r* = .510, 95% CIs, −0.293 – 0.288). For different urban habitat pairs, we detected a significant positive correlation between genetic distance and landscape difference for the UA_UP pairs, while other pair types showed no significant correlations (Figure 4h–j; UA_UP pairs, *r* = .439, 95% CIs, −0.377 to 0.336; UA_UR pairs, *r* = .235, 95% CIs, −0.341 to 0.336; UP_UR pairs, *r* = .363, 95% CIs, −0.370 to 0.376).

**FIGURE 5:**
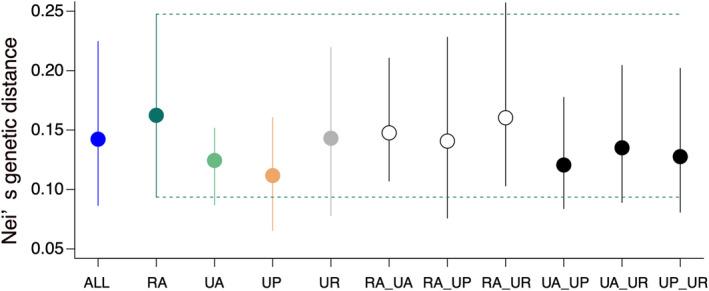
Comparison of Nei's genetic distance between pairs of *C. communis* populations within the RA, UA, UP, and UR habitat types, and between habitat types (indicated by underscores). ALL indicates all population pairs. White symbols represent paired populations between RA and various urban habitats; black symbols represent those between each different urban habitats. Dots are mean values for the population pairs; error bars indicate 95% confidence intervals (CIs). Dashed lines indicate the 95% CIs of the RA habitat population pair. RA, rural agricultural land; UA, urban agricultural land; UP, urban park; UR, urban roadside.

There were no significant differences in Nei's genetic distance among randomly sampled pairs of the same and different habitat types, as indicated by the overlapping 95% CIs of all paired habitat types (Figure 5).

**FIGURE 6:**
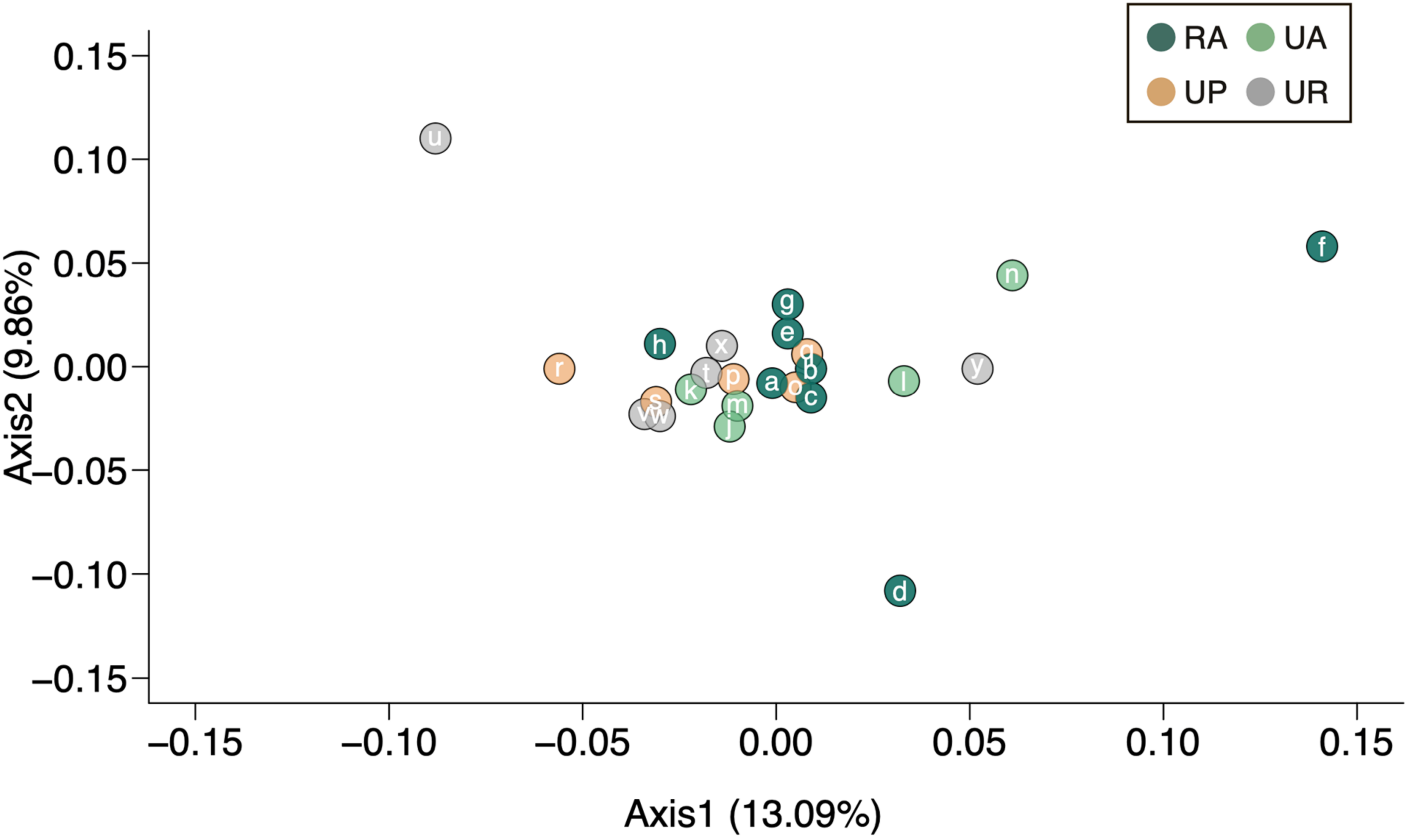
Principal coordinate analysis (PCoA) results for *C. comunis* populations based on Nei's genetic distance. Axes 1 and 2 account for 13.09% and 9.86% of the variance, respectively. Population *i* was excluded from this analysis due to insufficient data. RA, rural agricultural land; UA, urban agricultural land; UP, urban park; UR, urban roadside.

The PCoA based on Nei's genetic distances showed that approximately 22.95% of the total variation was explained by the first two principal coordinate axes, revealing no clear genetic differentiation among rural and urban habitat populations (Figure 6).

## 4 DISCUSSION

Our findings indicate that urbanisation‐induced habitat changes did not greatly influence genetic diversity in urban populations compared to rural populations of *C. communis*. Additionally, we observed no significant differences in genetic diversity and no clear genetic differentiation among the three urban habitat types (Figures 2 and 6, Figure S4), and no significant isolation‐by‐distance patterns for any habitat‐type pairs except for urban agricultural land and roadside population pairs (Figure 3, Figure S5). Meanwhile, difference in landscape configuration is suggested to promote genetic differentiation not only between urban–rural population pairs but also between urban agricultural land and park population pairs (Figure 4e–h). The results suggest that the reduction of forests due to increased developed lands may be an important factor affecting inter‐population genetic differentiation in *C. communis*. Our findings together suggest that urbanisation‐induced landscape changes have had some impacts on the genetic structure of this widely distributed annual species.

### 4.1 Genetic diversity and genetic structure among habitat types

Contrary to our expectation that urban populations have less genetic diversity than rural populations, urbanisation and subsequent landscape changes did not promote a loss of genetic diversity in *C. communis*. This is likely owing to extremely low genetic diversity of *C. communis* populations (Figure 2, Figure S4). We found that observed heterozygosity (*H*_O_) was extremely lower than expected heterozygosity (*H*_E_) (Figure 2a, Figure S4a). It should be noted that the MIG‐seq and RAD‐seq analysis results, which depend on genome‐wide SNPs, often exhibit lower *H*_O_ compared with *H*_E_ (Buckley et al., 2018; Nagasawa et al., 2020; Nakahama et al., 2021).

Our PCoA results demonstrated no clear genetic differentiation between urban and rural populations or among urban habitat types (Figure 6). Our findings are consistent with previous studies and reviews that have reported that urban development often does not significantly influence plant genetic diversity and/or structure (Hatanaka & Isagi, 2010; Johnson et al., 2018; Miles et al., 2019; Toma et al., 2015).

Instead, natural history appears to have played an important role in shaping the genetic patterns of this species, resulting in an absence of clearly urban‐induced effects. *C. communis* has a mixed mating system (Katsuhara & Ushimaru, 2019; Morita & Nigorikawa, 1999), and our results revealed relatively high inbreeding coefficients (*F*_IS_) and low *H*_O_ in both rural and urban populations (Figure 2c, Figure S4a). *H*_O_ values of some SSR loci were also lower than *H*_E_ in *C. communis* (Li et al., 2015). These findings suggest that the lack of genetic diversity differences among habitat types is attributable to a high population selfing ratio across the urban–rural gradient. Although pollinator visits to *C. communis* can vary among urban populations depending on the amount of surrounding green spaces, many urban populations appear to reproduce via self‐pollination (Ushimaru et al., 2014). A previous study reported that reproduction in *Viora grypoceras* occurred mainly by self‐pollination via cleistogamy, and that seed bank formation allowed this species to tolerate urban fragmentation (Toma et al., 2015); thus, selfing may be an important reproductive trait for avoiding the negative effects of urban fragmentation. By contrast, lower *H*_O_ and higher *F*_IS_ values were observed in urban populations of *Impatiens capensis* than in rural populations, which have higher outcrossing rates (Rivkin & Johnson, 2022), potentially indicating that selfing reduces genetic diversity in urban populations. Furthermore, the polyploidy in *C. communis*, as described by Fujishima (2003), might act as a mitigating factor against the negative impacts of urban development on genetic diversity (van Drunen & Johnson, 2022). To comprehensively understand the effects of selfing and polyploidy on genetic diversity and structure in *C. communis*, future studies should examine the pollination and mating systems of both urban and rural populations employing a combination of observations and genetic analyses.

### 4.2 Genetic differentiation via geographic distance and landscape difference in diverse urban habitats

Significant increases in genetic distances were observed between rural (RA) and urban agricultural land (UA) pairs, between RA and urban roadside (UR) pairs, and between UA and urban park (UP) pairs, as the surrounding landscape difference increased between the populations (Figure 4e,g). Meanwhile, no significant isolation by distance (IBD) was detected in these population pairs (Figure 3a,c). Our preliminary analyses revealed increasing differences in both developed land and forest areas significantly increased genetic differentiation, whereas differences in agricultural land areas did not influence genetic differentiation (Figure S6). In the case of *Sabatia angularis*, highly developed areas constrained gene flow, while forests and green corridors facilitated gene flow (Emel et al., 2021). Additionally, urban green corridors facilitated male reproductive success in comparison to non‐corridor areas in *Asclepias syriaca* (Breitbart et al., 2022). Visit frequencies of pollinators increased with increasing surrounding green spaces in *C. communis* (Ushimaru et al., 2014). Thus, the amount of surrounding green spaces likely facilitates seed and pollen dispersal which promotes inter‐population gene flows. UA and UR populations had smaller surrounding forest areas compared to RA and UP populations (Figure S1). Both habitat fragmentation by developed lands and limited forest spaces for urban agricultural land and roadside populations might enhance their genetic differentiation from rural and/or urban park populations in *C. communis*.

There was also no significant IBD detected between populations of the same habitat (Figure 3), and no relationships between genetic distance and landscape difference were found among populations within the same habitat (Figure 4a–d), suggesting that both Euclidean distance and landscape difference did not promote genetic differentiation within the same habitat‐type populations.

No correlation between genetic distance and geographic distance or landscape was found for the UP and UR population pairs (Figures 3 and 4i,j, Figure S5b), and the absence of IBD in urban herb populations is also reported by the previous studies (Fukano et al., 2023; Johnson et al., 2018; Lu et al., 2022). It was indicated that founder effects and population isolation in urban habitats did not significantly influence genetic differentiation in this study species. One factor that may prevent the development of IBD patterns is unintentional dispersal via human activities such as the creation of traffic road networks, as demonstrated in the invasive plant *Ambrosia artemisiifolia* in urban areas (Lu et al., 2022). Accidental dispersal by footwear and by importing soil may have occurred during the initial development of parks and roadside green spaces. We found that most urban park and roadside populations had genetic patterns that were similar to urban and rural agricultural land populations, which suggests that most urban park and roadside populations were established through the rapid recovery of *C. communis* individuals distributed near the parks and roads within our study area.

Our knowledge of the genetic structures of plants distributed across diverse urban habitat types remains limited. To understand the effects of habitat diversification and historical habitat fragmentation and formation processes on the genetic structure of urban plant populations, further data are needed to represent a wider range of urban habitat characteristics and the genetic compositions of species with diverse mating systems.

We found significant positive correlations between genetic and geographic distance among UA and UR pairs (Figure S5), whereas no significant correlation between genetic distance and landscape difference was found among the pairs (Figure 4i). However, the underlying reasons for these observed patterns remain unclear due to insufficient data. Future studies should confirm these patterns and clarify their mechanisms by adding study populations and assessing the connectivity of urban green spaces.

## 5 CONCLUSION

Our findings reveal that urbanisation neither led to a loss of genetic diversity nor largely altered the degree of genetic differentiation in various urban habitat types, suggesting the tolerance of *C. communis* against habitat fragmentation and environmental changes conferred by its mixed mating system and high ability to adapt to ruderal conditions. We found, however, that differences in landscape configuration promoted genetic differentiation between rural and urban agricultural land and roadside populations as well as between urban agricultural land and park populations. These findings suggest that a decrease in forests due to increased developed lands around habitats may contribute to the genetic differentiation of urban agricultural land and roadside populations to some extent.

Limitations of this study include its small geographic scale compared to previous studies (Johnson et al., 2018; Santangelo, Ness, et al., 2022; Yakub & Tiffin, 2017). As *C. communis* has invaded multiple countries (iNaturalist, https://www.inaturalist.org/), it is an ideal model species for exploring the effects of urbanisation on genetic diversity and differentiation between native and non‐native areas (Lu et al., 2022; Santangelo, Ness, et al., 2022). Further investigations at larger spatial scales are needed to obtain a comprehensive understanding of the urban evolutionary dynamics of this species.

There is limited information on phenotypic trait differentiation along urban–rural gradients available for *C. communis* (Taichi & Ushimaru, 2024; Ushimaru et al., 2014). To understand the relationship between genetic and phenotypic trait adaptation in this species, we propose conducting trait measurements in common garden and reciprocal transplant experiments (e.g., Gorton et al., 2018) to determine whether diverse urban habitat populations are forced to adapt to local environments or can survive in a range of urban habitats.

## AUTHOR CONTRIBUTIONS

**Nakata Taichi:** Conceptualization (lead); data curation (lead); formal analysis (equal); funding acquisition (equal); investigation (equal); resources (equal); visualization (equal); writing – original draft (equal); writing – review and editing (equal). **Naoyuki Nakahama:** Data curation (supporting); formal analysis (equal); methodology (lead); resources (equal); visualization (equal); writing – review and editing (equal). **Nobuko Ohmido:** Investigation (supporting); resources (supporting); writing – review and editing (equal). **Atushi Ushimaru:** Conceptualization (equal); formal analysis (equal); funding acquisition (equal); resources (supporting); supervision (lead); visualization (equal); writing – original draft (equal); writing – review and editing (equal).

## CONFLICT OF INTEREST STATEMENT

The authors declare no conflict of interest.

## Supporting information


Data S1.

## Data Availability

Data_ECE‐2023‐12‐02140.xlsx were row data to use in the analysis of our MS. These data included this URL (https://zenodo.org/records/10295101).
