# Supplementary material for: Habitat diversification associated with urban development has a little effect on genetic structure in the annual native plant Commelina communis in an East Asian megacity
Source: Ecol Evol. 2024 Feb 21;14(2):e10975. doi: 10.1002/ece3.10975 (PMC10880129; doi:10.1002/ece3.10975)
Supplement: Supplementary file 1 — Data S1. [file ECE3-14-e10975-s001.zip › MS_Apendix_revision1204.docx]

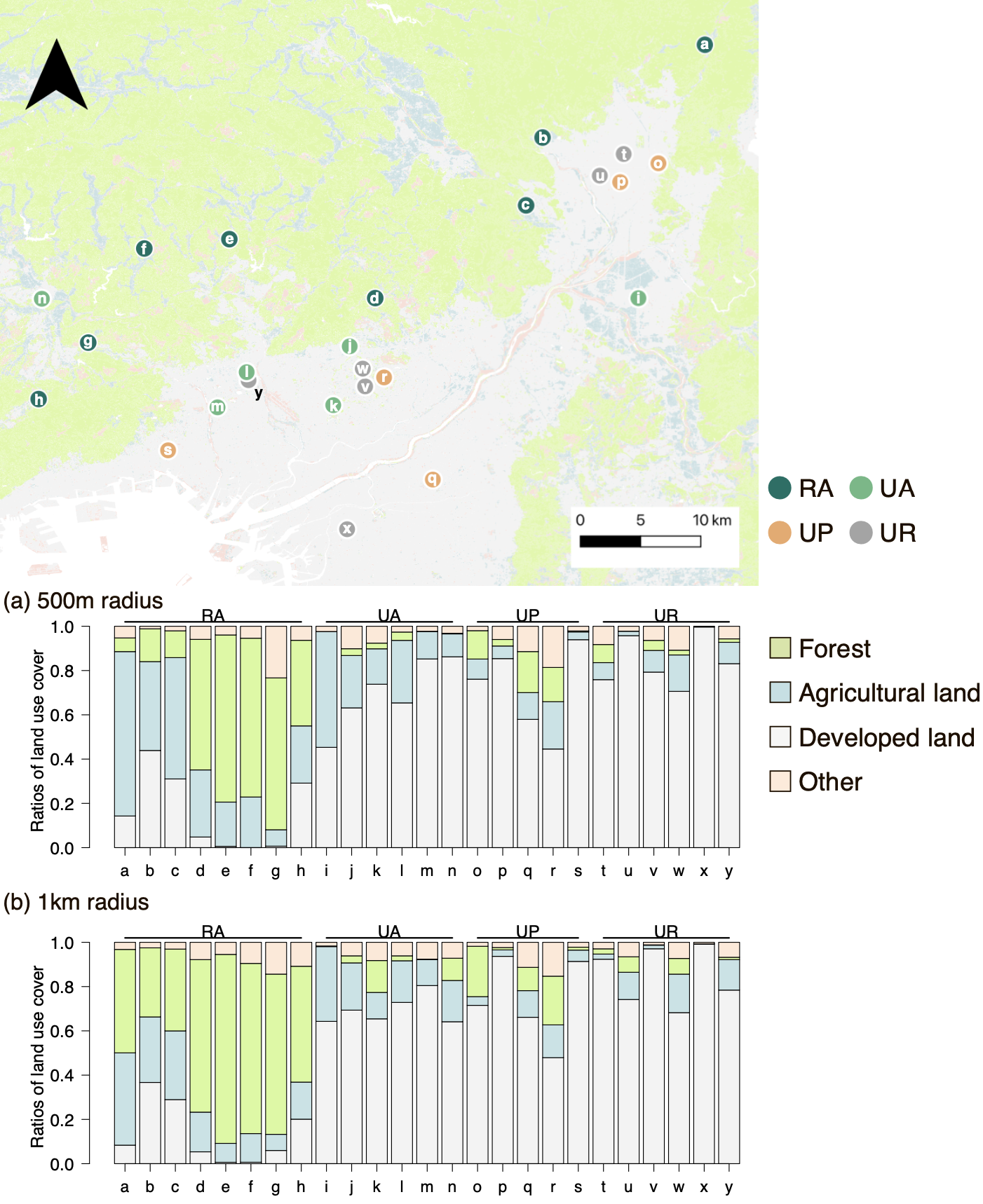


Figure S1 Map of the 25 study sites in the Kyoto-Osaka-Kobe metropolitan area. Grey, light green, light blue, and white areas are developed lands, forests, agricultural lands, and water, respectively. Bar plots indicate the ratios of four land uses (developed lands, agricultural lands, forests, and others) within 500m and 1km radii from the centre of each site.


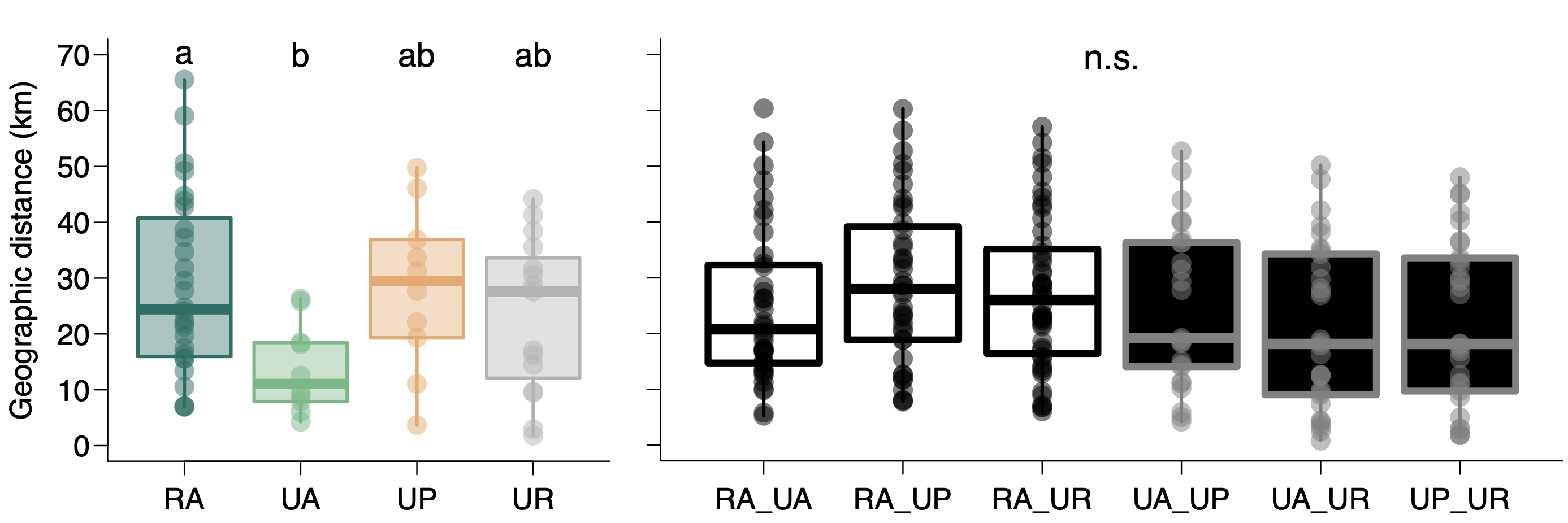


Figure S2 Comparison of inter-site geographical distances for each habitat type. RA: rural agricultural land, UA: urban agricultural land, UP: urban park, UR: urban roadside, RA_UA, RA_UP, RA_UR: pairs of RA and three urban habitat types, UA_UP, UA_UR, UP_UR: different urban habitat pairs. Boxplots represent medians (bold horizontal lines). Each dot represents the value for each population plot. Different letters indicate significant (*P* < 0.05) differences among habitat types based on the Tukey method.


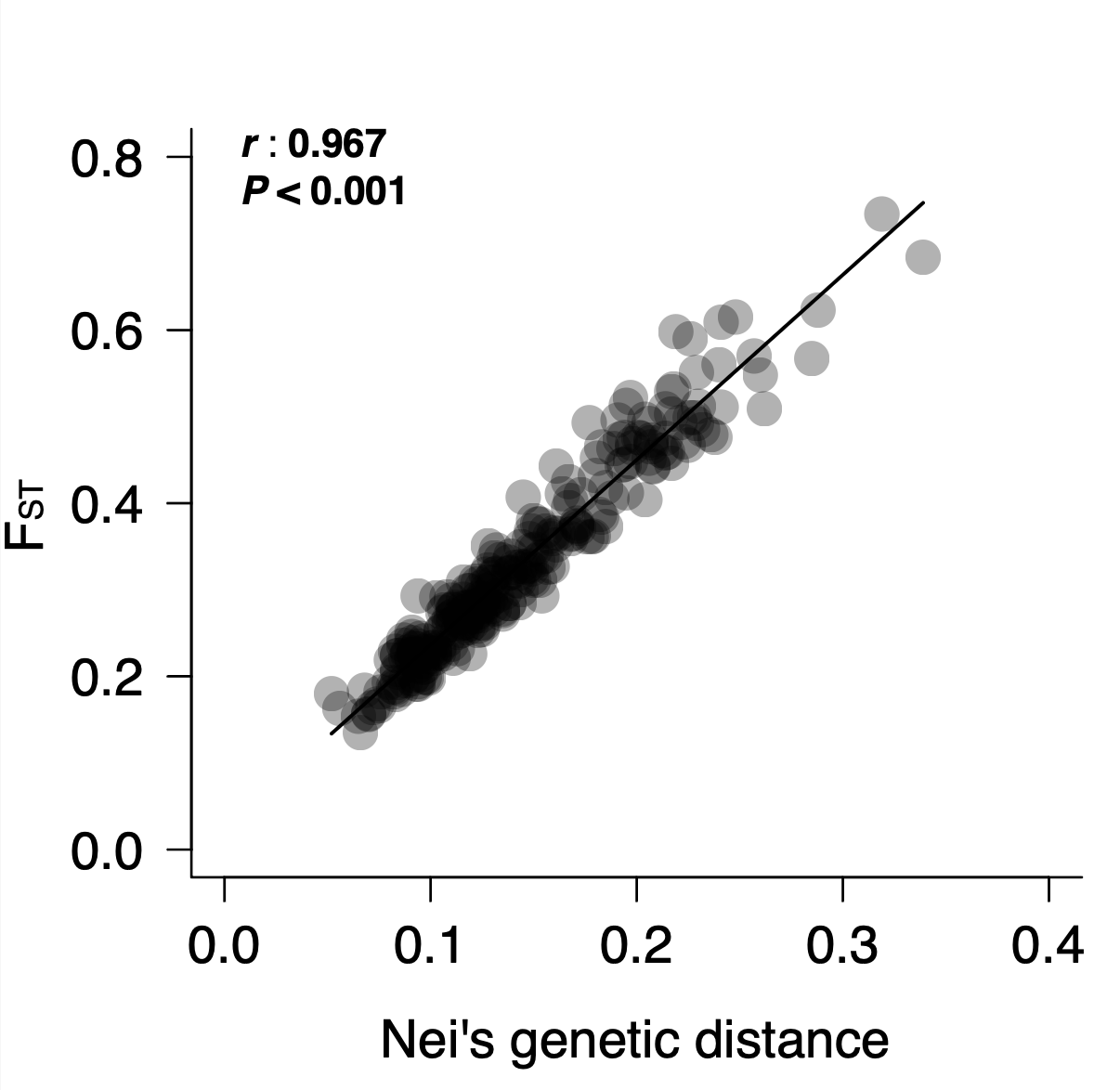


Fig. S3 Relationship between Nei's genetic distance and F_ST_. A regression line for the relationship between the indices was drawn using the estimated coefficients from the Mantel test with 9999 permutations.


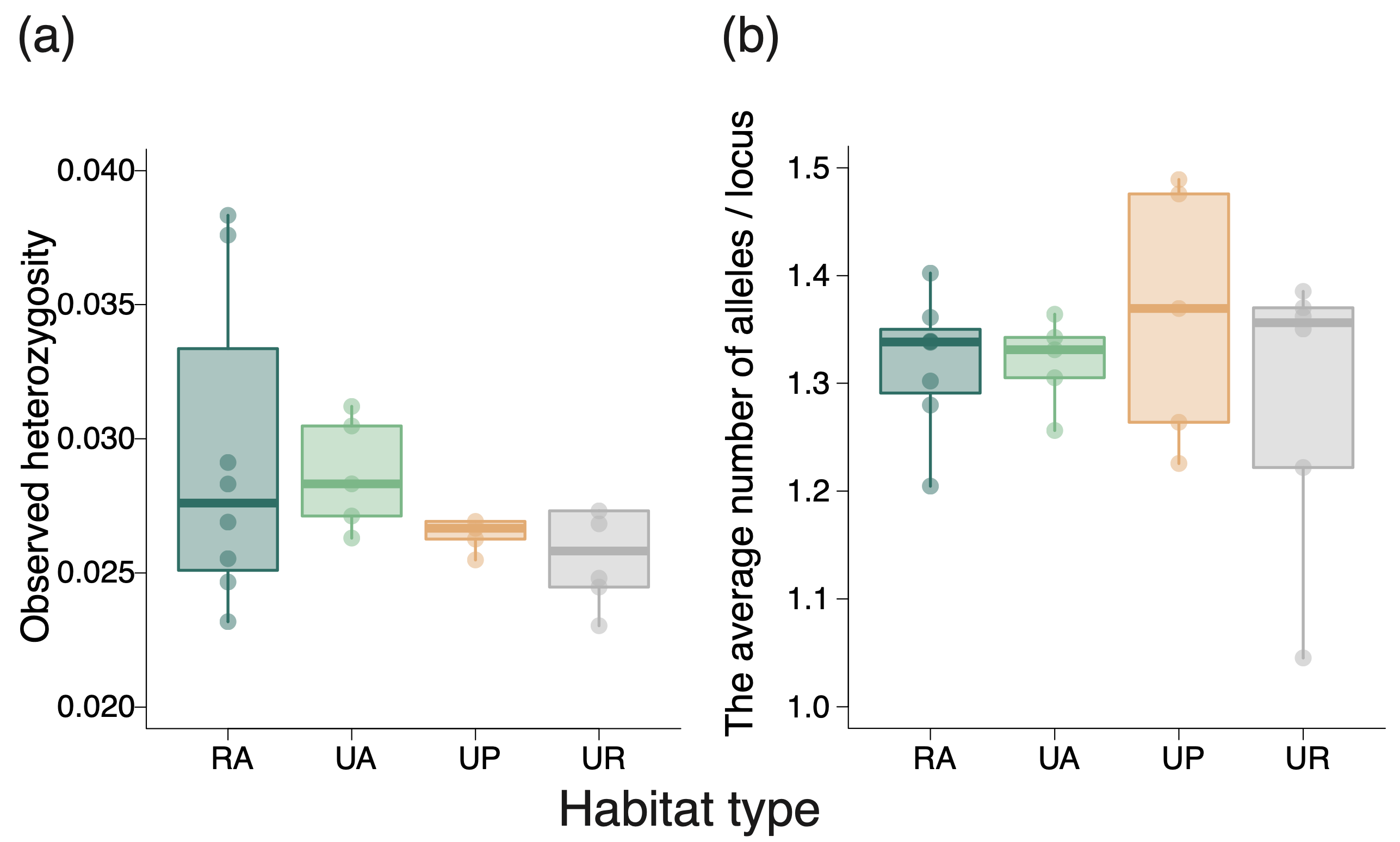


Figure. S4 Comparisons of genetic diversity measurements: (a) observed heterozygosity and (b) the average number of alleles/ locus for four habitat types. RA: rural agricultural land, UA: urban agricultural land, UP: urban park, UR: urban roadside. Boxplots represent medians (bold horizontal lines). Each dot represents the value for each population plot.


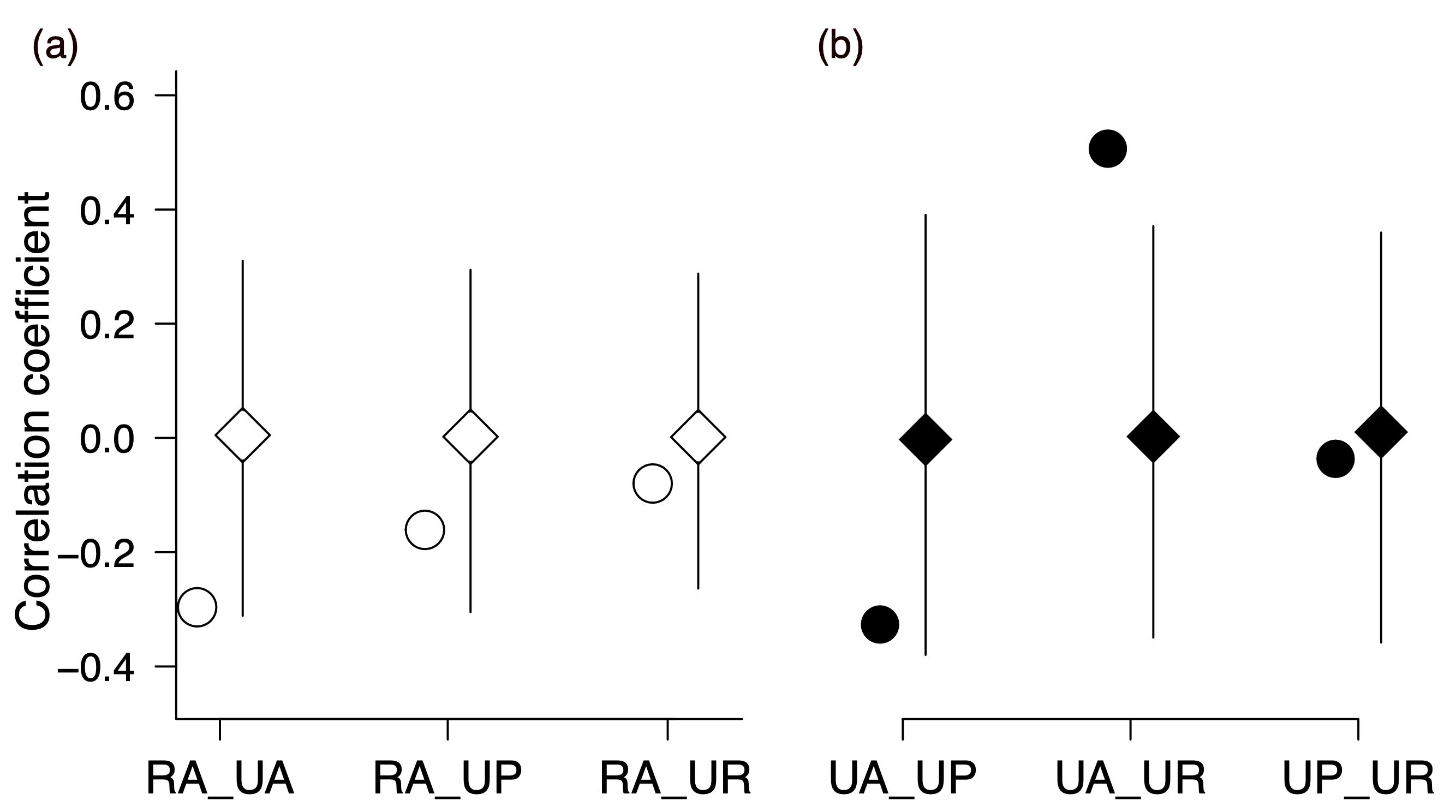


Fig. S5 Results of permutation tests for correlation coefficients between genetic and geographic distances in different pairs of habitat types: (a) pairs of RA and three urban habitat types, (b) different urban habitat pairs. The circles and squares show the observed correlation coefficients and the expected mean values of correlation coefficients, which were calculated from 1000 permutations with 95% CIs, respectively.


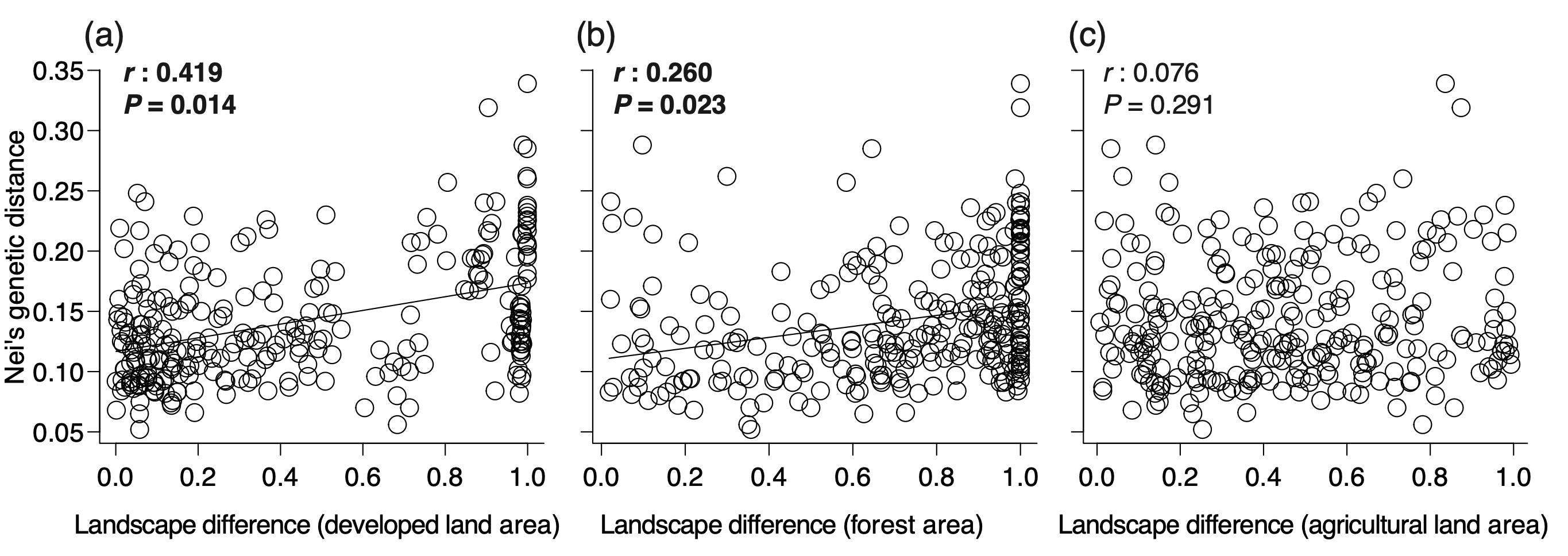


Figure S6 Relationships between Nei’s genetic distance and landscape difference: (a) developed land area, (b) forest area, (c) agricultural land area. Regression lines for the relationship between the variables were drawn using the estimated coefficients from the general linear model analyses.
